# Mechanism Analysis of Zuogui and Yougui Pills on Diabetic Nephropathy Through Transcriptional Regulatory Networks of HIF1A and PPARA

**DOI:** 10.1002/fsn3.70317

**Published:** 2025-05-23

**Authors:** Liansheng Qiao, Xiaopeng Zhao, Anlei Yuan, Chaoqun Liu, Zewen Wang, Xiaoqian Huo, Shijie Bi, Jiaye Tian, Bin Yu, Zhaozhou Lin, Yanling Zhang, Jiwang Zhang

**Affiliations:** ^1^ Key Laboratory of TCM‐Information Engineer of State Administration of TCM, School of Chinese Materia Medica Beijing University of Chinese Medicine Beijing China; ^2^ Beijing Tongrentang Co., Ltd. Beijing China

**Keywords:** diabetic nephropathy, HIF1A, PPARA, traditional Chinese medicine, transcriptional regulatory network

## Abstract

Diabetic nephropathy, a serious diabetes complication, lacks effective treatments. Traditional Chinese medicine Zuogui Pill (ZGP) and Yougui Pill (YGP) have a definite clinical adjunctive effect on diabetic nephropathy. Given their similar compositions, studying their shared mechanisms could provide novel perspectives into discovering therapeutic targets for diabetic nephropathy. Extraction of ZGP (EZP) and YGP (EYP) was prepared by in vitro digestion. EZP and EYP inhibited the gene expression of *FN1* and *MMP9* in a renal fibrosis cell model. A transcriptional regulatory network revealed EZP and EYP have similar mechanisms, with HIF1A as a key transcription factor. In an insulin resistance cell model, EZP and EYP led to the decrease in glucose consumption. A transcriptional regulatory network suggested that EZP and EYP have different regulatory panels, but PPARA was the common transcriptional factor. *CA9* and *PDK1*, the downstream genes of HIF1A, and *PDK4*, the downstream gene of PPARA, were activated by both EZP and EYP, which showed the potential new targets of diabetic nephropathy. A total of 42 compounds from EZP and EYP were screened as the potential mediators of HIF1AN and EGLN1, interacted proteins and common targets of HIF1A. A total of 8 compounds, including verbascoside, were screened as the potential PPARA agonists based on molecular docking. Verbascoside improved the decrease in glucose consumption. The study clarified the mechanism of the ZGP and YGP by regulating the transcriptional regulatory network of HIF1A and PPARA and provided new ideas for the discovery of potential targets for the treatment of diabetic nephropathy and the development of natural nutrients.

## Graphical abstract

The shared mechanisms of Zuogui Pill (ZGP) and Yougui Pill (YGP) for treating diabetic nephropathy is HIF1A‐CA9‐PDK1 and PPARA‐PDK4. ZGP and YGP inhibited renal fibrosis and relieved the decrease in glucose consumption through transcriptional regulatory network of HIF1A and PPARA. The active ingredients of ZGP and YGP were discovered for regulating HIF1A and PPARA.

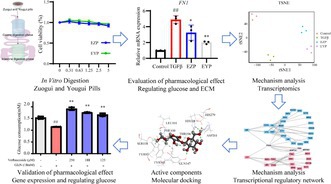

## 1 Introduction

Diabetic nephropathy is a significant complication associated with diabetes mellitus. As of 2021, the global prevalence of diabetes has reached 537 million individuals, with an estimated 20%–50% of these patients likely to develop diabetic nephropathy (Hoogeveen 2022). Currently, there is a lack of therapeutic drugs for diabetic nephropathy. Approved therapeutic targets for diabetic nephropathy include sodium‐glucose cotransporter‐2, mineralocorticoid receptor, and glucagon‐like peptide‐1 receptor; however, the efficacy of these approved drugs remains suboptimal and warrants further enhancement (Tuttle et al. 2022). Renal injury and metabolic disorder are two important pathological changes in diabetic nephropathy (Agarwal 2021). Renal fibrosis is one of the most essential pathological processes of renal injury in diabetic nephropathy. The liver is the main organ for the body to store and metabolize glucose and lipids. Like diabetes, diabetic nephropathy also has abnormal glucose metabolism and insulin resistance in the liver (Dong et al. 2024). Diabetic nephropathy is involved in multiple pathological processes, and new therapeutic drugs and targets are urgently needed for diabetic nephropathy.

Traditional Chinese medicine (TCM) has a definite effect on diabetic nephropathy (Tang et al. 2021). At present, more than 20 formulae, such as Liuwei Dihuang Pill, Shenqi Dihuang Decoction, and so on, have proved to have a good effect on diabetic nephropathy, mainly improving blood glucose, urinary protein, serum creatinine, blood urea nitrogen, and so on (Ma et al. 2023). However, the targets and active ingredients are not very clear.

Zuogui Pill (ZGP) and Yougui Pill (YGP) are classic tonic formulae used in clinics by TCM, which were first presented by Jingyue Quanshu. ZGP and YGP have the advantage of treating metabolic disorders (Zhang, Hu, et al. 2024), kidney diseases, and osteoporosis (Li et al. 2018), male diseases (Feng et al. 2022), or gynecological diseases (Zhong et al. 2022) in clinics. Diabetic nephropathy is a typical comprehensive disease of metabolic disorders and kidney disease, and ZGP and YGP are commonly used in the clinical treatment of diabetic nephropathy. Pharmacological studies have shown that ZGP is effective in the treatment of diabetic nephropathy (Zhu et al. 2022), gestational diabetes mellitus (Wang et al. 2014), and type 2 diabetic osteoporosis (Shi et al. 2023). The pharmacological studies of YGP focused on improving renal function in unilateral ureteral obstruction‐induced renal tubulointerstitial fibrosis in rats (Wang et al. 2015) and hydrocortisone‐induced rat models (Chen et al. 2019). However, the mechanism of anti‐diabetic nephropathy of ZGP and YGP is still unclear.

ZGP and YGP have similar pharmacological effects based on their similar composition. ZGP consists of 8 TCMs, and YGP consists of 10 TCMs. Six TCMs are both components of ZGP and YGP, including *Rehmanniae Radix Praeparata* (Shudihuang), *Dioscoreae Rhizoma* (Shanyao), *Lycii Fructus* (Gouqizi), *Cuscutae Semen* (Tusizi), *Corni Fructus* (Shanzhuyu), and *Testudinis Carapacis Et Plastri Colla* (Lujiaojiao). The unique TCMs of ZGP are *Cyathulae Radix* (Chuanniuxi) and *Testudinis Carapacis Et Plastri Colla* (Guijiajiao). The unique TCMs of YGP are *Aconiti Lateralis Radix Praeparata* (Fuzi), *Cinnamomi Cortex* (Rougui), *Angelicae Sinensis Radix* (Danggui), and *Eucommiae Cortex* (Duzhong). Therefore, ZGP and YGP might have similar mechanisms and be studied together. ZGP and YGP provide suitable research materials to study their unique and common mechanism of action in treating diabetic nephropathy. ZGP and YGP possess similar compositions and are both utilized in the treatment of diabetic nephropathy. However, in TCM clinical practice, ZGP and YGP are employed to treat diabetic nephropathy associated with distinct syndromes. Consequently, we hypothesized that their shared mechanism may underlie their efficacy in treating diabetic nephropathy, while their different mechanisms may reflect the different syndromes. To identify potential therapeutic targets for diabetic nephropathy, our study concentrated on elucidating the common mechanisms of these two formulae.

The purpose of this study was to uncover the anti‐diabetic nephropathy mechanism by ZGP and YGP and explore the novel therapeutic targets for diabetic nephropathy. Extraction of ZGP (EZP) and extraction of YGP (EYP) were prepared by in vitro digestion. Transforming growth factor β (TGFβ)‐induced HK2 and glucosamine (GlcN)‐induced HepG2 cells were utilized to validate the pharmacological effect of renal fibrosis and insulin resistance regulated by the two formulae, respectively. Transcriptomics analysis combined with transcriptional regulation networks was used to analyze the signal pathways and key transcription factors regulated by ZGP and YGP for diabetic nephropathy. Downstream gene expression of signal pathways and transcription factors was further validated through qRT‐PCR. The active ingredients of ZGP and YGP were discovered by combining molecular docking and cell experiments.

## 2 Materials and Methods

### 2.1 Materials

ZGP and EYP were purchased from Tongrentang Medicine Corporation Ltd. (Beijing, China). HepG2 cells were purchased from ATCC. HK2 cells were provided by Procell Life Science & Technology Co. Ltd. (Wuhan, China). TGFβ was obtained from PeproTech. GlcN was purchased from Beyotime.

### 2.2 In Vitro Digestion

EZP and EYP were prepared by in vitro digestion. Electrolyte stock solutions of simulated gastric fluids (SGF) and simulated intestinal fluids (SIF) were prepared as a standardized procedure (Minekus et al. 2014). In the gastric digestion phase, ZGP or EYP (10 g) was mixed with pepsin (2000 U/mL), SGF, CaCl_2_, and water at pH 3.0 and incubated at 37°C for 2 h. In the intestinal digestion phase, the gastric digests were mixed with trypsin (100 U/mL), SIF, CaCl_2_, bile, and water at pH 7.0 and incubated at 37°C for 2 h. An extraction mimic was also prepared without adding formula powder under the same digestion conditions to eliminate interference from the digestion components. The samples were then centrifuged at 10,000 rpm for 10 min. The supernatants were freeze‐dried and dissolved in PBS at 100 mg/mL.

### 2.3 Cell Culture

HK2 cells were cultured in MEM supplemented with 10% FBS and 1% penicillin‐streptomycin (PS). HepG2 cells were cultured in DMEM with 10% FBS and 1% PS. All cell cultures were maintained at 37°C and 5% CO_2_.

### 2.4 Cell Viability Assay

Approximately 0.5 × 10^4^ HK2 cells per well were seeded into 96‐well plates and incubated overnight. Subsequently, the cells were treated with five different concentrations of EZP and EYP for 72 h. Similarly, 2 × 10^4^ HepG2 cells per well were seeded into 96‐well plates and treated with EZP and EYP for 24 h. Following treatment, the medium was removed, and MTT (0.5 mg/mL) was added to the cells and incubated for 4 h. The reaction was terminated using DMSO, and the absorbance at 490 nm was measured using a FlexStation 3.

### 2.5 TGFβ‐Induced HK2 Cells

HK2 cells were seeded into 6‐well plates at a density of 2 × 10^5^ cells per well and incubated overnight. Subsequently, the cells were treated with TGFβ prepared in MEM without serum for 72 h to establish a renal fibrosis cell model. EZP and EYP were co‐administered with TGFβ for the same duration to assess the effects of the two formulae.

### 2.6 qRT–PCR

Total RNA was isolated from the cells utilizing a TRIzol reagent. Subsequently, cDNA was synthesized from 1 μg of RNA using the FastKing cDNA reverse transcription kit (Tiangen). qRT–PCR was performed using SYBR Premix Ex Taq II (Takara Inc., Japan) along with specific gene primers (Table S1). The thermal cycling protocol was as follows: 95°C for 30 s, followed by 50 cycles of 95°C for 5 s and 60°C for 34 s. Gene expression levels were quantified to assess the fold change relative to the expression of *GAPDH*.

### 2.7 RNA‐Seq

Total RNA from cell models treated with EZP and EYP was harvested and extracted using a TRIzol reagent. The RNA quality was assessed utilizing the Bioanalyzer 2100 system (Agilent Technologies, CA, USA). Subsequently, cDNA libraries were constructed and sequenced on the Illumina NovaSeq platform. Raw sequencing reads were aligned to the reference genome using Hisat2 v2.0.5. The analysis of t‐distributed stochastic neighbor embedding (t‐SNE) and principal component analysis (PCA) were conducted utilizing the R software package. Differentially expressed genes (DEGs) were identified using DESeq2.

### 2.8 DEGs and Pathway Enrichment

Volcano and heatmap analysis of DEGs was performed using the R package. Pathways were further enriched with DEGs according to KEGG, Reactome, and GO in DAVID (https://david.ncifcrf.gov/). Diabetic nephropathy‐related genes were collected from the disease‐gene databases CTD (Davis et al. 2022), MalaCards (Rappaport et al. 2016) and DisGeNET (Piñero et al. 2017).

### 2.9 Transcriptional Regulatory Network Analysis

Transcriptional factors regulated by EZP and EYP were enriched using DEGs by TRRUST (Han et al. 2018). The transcriptional regulatory network of the transcriptional factors and downstream genes was visualized by Cytoscape. Protein–protein interaction of key transcriptional factors was obtained from the STRING database, utilizing text mining, experiments, and co‐expression with a confidence score of 0.9. The analysis was restricted to a maximum of 10 interactors from the first shell. The protein interaction network (PIN) of key transcriptional factors was constructed using Cytoscape.

### 2.10 GlcN‐Induced HepG2 Cells

HepG2 cells were seeded in 96‐well plates at a density of 2 × 10^4^ cells per well. Following a 24‐h attachment period, the cells were treated with 18 mM GlcN in serum‐free DMEM medium with low glucose to induce a reduction in glucose consumption. After an 18‐h induction period, varying concentrations of EZP and EYP were administered for an additional 24 h. The glucose concentration in the cell supernatant was subsequently measured using the GOD‐POD method, with glucose consumption being quantified at an absorbance of 505 nm using a FlexStation 3.

### 2.11 Molecular Docking

A set of 831 compounds were collected from TCMs in ZGP and YGP according to TCMBank (Lv et al. 2023) and HIT (Yan et al. 2022), excluding Lujiaojiao and Guijiajiao. The three‐dimensional structures of these compounds were generated, and their energies were minimized using Discovery Studio. The crystal structures of HIF1AN [1H2M (Elkins et al. 2003)], EGLN1 [4BQW (Chowdhury et al. 2013)], and PPARA [3KDU (Li et al. 2010)] were downloaded from PDB. The binding sites of these three proteins were identified based on initial ligands complexed in crystal structures. The CDOCKER algorithm was employed for molecular docking. 90% of ‐CDOCKER interaction energy of initial ligands was utilized as the cut‐off for screening the active compounds that interacted with these three proteins.

### 2.12 Statistical Analysis

The results were presented as the mean ± standard deviation (SD) and were analyzed using Student's *t*‐test. Statistical analyses were conducted utilizing GraphPad Prism software. A *p*‐value of less than 0.05 was considered to denote statistical significance.

## 3 Results

### 3.1 Alleviation of Renal Fibrosis by EZP and EYP in TGFβ‐Induced HK2 Cells

**FIGURE 1:**
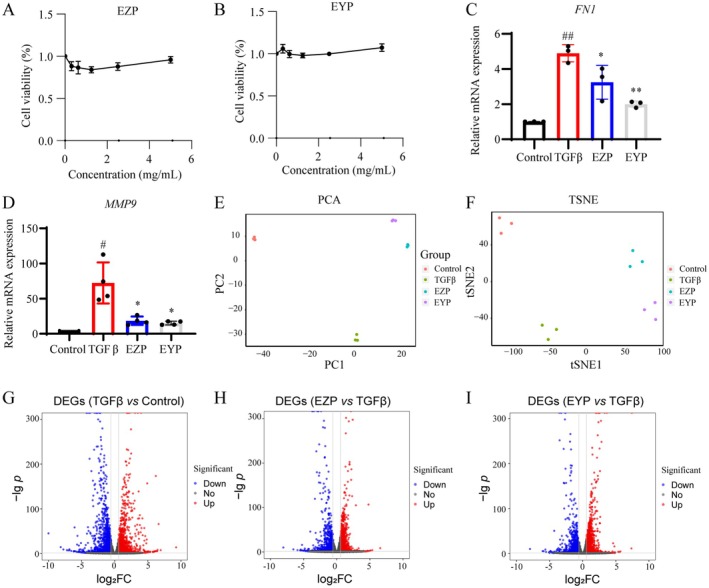
EZP and EYP reduced renal fibrosis in TGFβ‐induced HK2 cells. Cell viability of HK2 was assessed with varying EZP (A) and EYP (B) concentrations for 72 h. Gene expression of *FN1* (C) and *MMP9* (D) regulated by EZP and EYP for 72 h. ## represented *p* < 0.01 vs. control. # represented *p* < 0.05 vs. control. **p < 0.01 vs. TGFβ. *p < 0.05 vs. TGFβ. PCA (E) and t‐SNE (F) analysis for control, TGFβ, EZP, and EYP groups. Volcano plots of DEGs were created for comparisons of TGFβ vs. control (G), EZP vs. TGFβ (H), and EYP vs. TGFβ (I) using |log_2_FC| > 0.58 and *p* < 0.05 as the cut‐off.

To investigate the molecular mechanisms by which EZP and EYP mitigate the pathological alterations associated with diabetic nephropathy in renal tubular epithelial cells, TGFβ‐induced HK2 cells were utilized for in vitro studies. MTT assay was performed to evaluate the cytotoxic effects of EZP and EYP, followed by further mechanistic studies. HK2 cells were treated with the indicated concentrations of EZP or EYP (0.3125 to 5 mg/mL) for 72 h, and the results indicated that both EZP (Figure 1A) and EYP (Figure 1B) exhibited minimal cytotoxicity in HK2 cells. Fibronectin 1 (FN1) and matrix metalloproteinase 9 (MMP9) were the typical extracellular matrix (ECM) and pathological biomarkers of renal fibrosis. In order to investigate the possible role of EZP and EYP in the regulation of the ECM, HK2 cells were treated with TGFβ in either EZP (2.5 mg/mL) or EYP (5 mg/mL). It was observed that both EZP and EYP significantly diminished TGFβ‐mediated gene expression of *FN1* and *MMP9* at 72 h (Figure 1C,D). EZP and EYP demonstrated a promising impact on the modulation of renal fibrosis.

To further explore the molecular mechanisms underlying the anti‐renal fibrosis effects of EZP and EYP, RNA‐seq analysis was conducted on HK2 cells. The HK2 cells in the control group were treated with an extraction mimic. The TGFβ‐induced HK2 cells were treated with the extraction mimic, EZP (2.5 mg/mL) or EYP (5 mg/mL) for 72 h, resulting in the designation of the TGFβ group, EZP group, and EYP group, respectively. Notably, the TGFβ group showed the most significant deviation from the control group. In contrast, the EZP and EYP groups partially reversed the gene expression profile characteristic of the TGFβ group, as indicated by PCA (Figure 1E) and t‐SNE (Figure 1F) analysis. The findings of this study indicated that EZP and EYP mitigated the pathological alterations induced by TGFβ, such as renal fibrosis, in HK2 cells.

### 3.2 HIF1A and PPARA were the Key Transcriptional Factors Regulated by EZP and EYP in Renal Fibrosis

DEGs were analyzed to elucidate the anti‐renal fibrosis mechanism of EZP and EYP. We identified 3336 DEGs when comparing the TGFβ group to the control group. Among these, 1678 were upregulated (red), and 1658 were downregulated (blue, Figure 1G). In the comparison between the EZP group and the TGFβ group, 2478 DEGs were detected, with 1133 upregulated and 1345 downregulated (Figure 1H). Additionally, in the EYP group relative to the TGFβ group, 2856 DEGs were detected, with 1428 upregulated and 1437 downregulated (Figure 1I).

**FIGURE 2:**
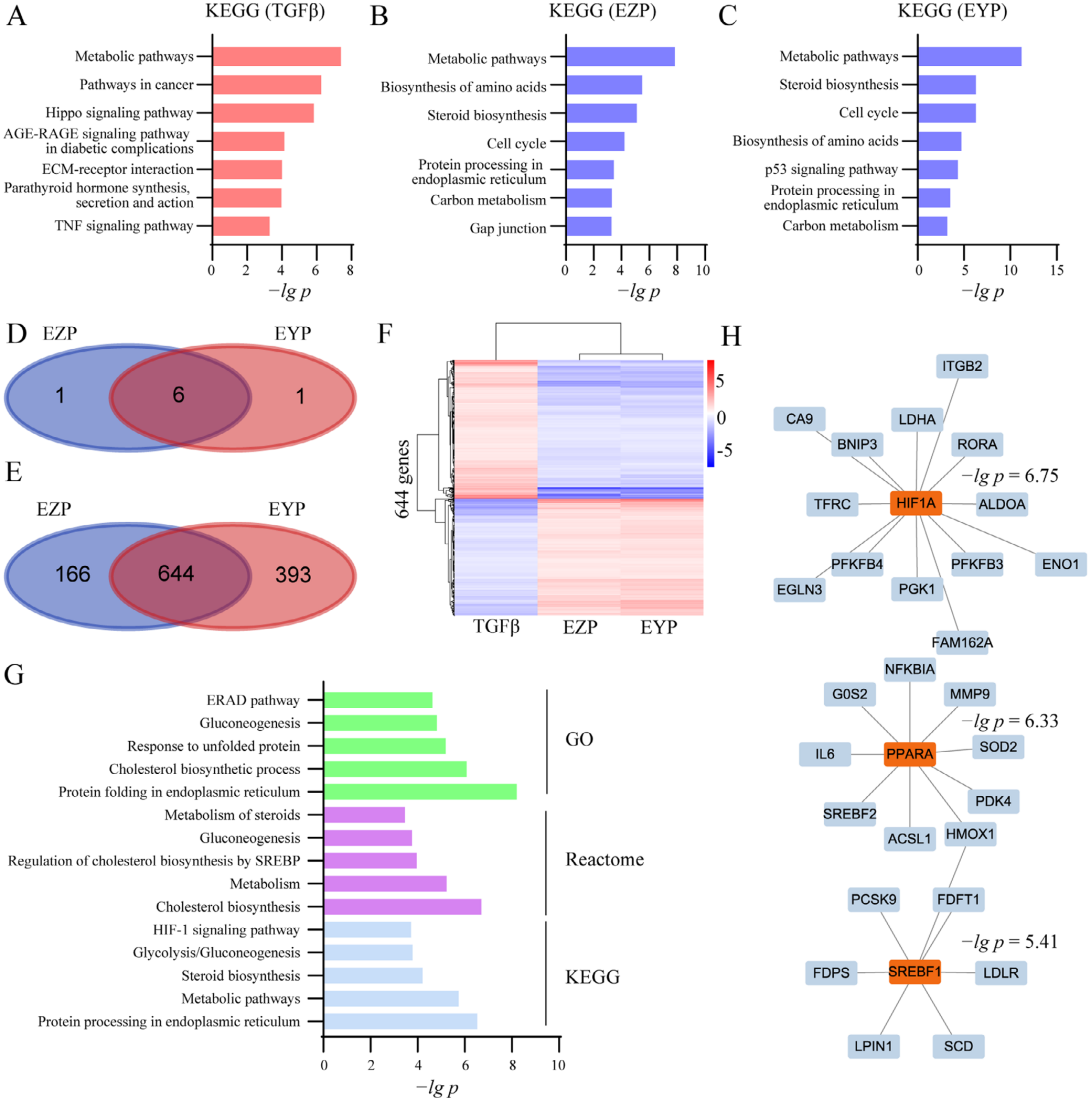
EZP and EYP regulated HIF1A and PPARA transcriptional regulatory networks. Pathway enrichment based on KEGG for TGFβ (A), EZP (B), and EYP (C) group. Veen analysis of enriched pathways (D) and opposite DEGs (E) for EZP and EYP. (F) Heatmap of opposite DEGs for TGFβ, EZP, and EYP based on hierarchical clustering. (G) Pathway enrichment of opposite DEGs. (H) Transcriptional regulatory network of common opposite DEGs regulated by EZP and EYP. Transcription factors are orange in color, while downstream genes are blue.

Pathway enrichment analysis based on KEGG was performed on DEGs to uncover TGFβ‐, EZP‐, and EYP‐mediated signaling pathways. The top seven TGFβ‐, EZP‐, and EYP‐regulated pathways are shown in Figure 2A–C. Signal pathways closely related to diabetic nephropathy were enriched in TGFβ‐induced HK2 cells, including metabolic pathways, AGE‐RAGE signaling pathway in diabetic complications, and ECM‐receptor interaction. These indicated that TGFβ‐induced HK2 cells can simulate the pathological changes of diabetic nephropathy. EZP and EYP regulated six similar metabolic signal pathways in TGFβ‐induced HK2 cells, including metabolic pathways, biosynthesis of amino acids, steroid biosynthesis, cell cycle, protein processing in endoplasmic reticulum, and carbon metabolism (Figure 2D). It suggested that EZP and EYP had a similar anti‐renal fibrosis mechanism.

Subsequently, the DEGs in the TGFβ group that can be reversed by the EZP or EYP group are named opposite DEGs, and these genes are analyzed in the two formulae, respectively. The EZP group identified 810 opposite DEGs, the EYP identified 1037 opposite DEGs, and their intersection is 644 opposite DEGs (Figure 2E). 295 genes were downregulated by TGFβ and upregulated by EZP or EYP. Additionally, 349 genes were upregulated by TGFβ and downregulated by EZP or EYP (Figure 2F). Pathway enrichment was conducted for opposite DEGs of EZP and EYP based on GO, Reactome, and KEGG. The top 5 pathways are shown in Figure 2G. Numerous glucose and lipid metabolic pathways were regulated by EZP and EYP, including gluconeogenesis and cholesterol biosynthetic process from GO, cholesterol biosynthesis, metabolism, regulation of cholesterol biosynthesis by SREBP (SREBF), and gluconeogenesis from Reactome and metabolic pathways and glycolysis/gluconeogenesis from KEGG. Meanwhile, the HIF‐1 signaling pathway was also enriched by opposite DEGs of EZP and EYP. These suggested glycolipid metabolism and hypoxia pathways were the core pathways for regulating renal fibrosis by EZP and EYP.

The transcriptional regulatory network was constructed to reveal the anti‐renal fibrosis mechanism of EZP and EYP. Transcriptional factors were enriched based on opposite DEGs of EZP and EYP. The top 3 transcriptional factors and 28 of their downstream direct regulatory genes were utilized to construct a transcriptional regulatory network for EZP and EYP (Figure 2H). These results were consistent with pathway enrichment, and at the same time, they better reflected the mechanism of action of the two formulae than the pathway enrichment. HIF1A, PPARA, and SREBF1 were key transcriptional factors for the treatment of diabetic nephropathy by EZP and EYP in renal function.

### 3.3 The Mitigation of Insulin Resistance by EZP and EYP in GlcN‐Induced HepG2 Cells

**FIGURE 3:**
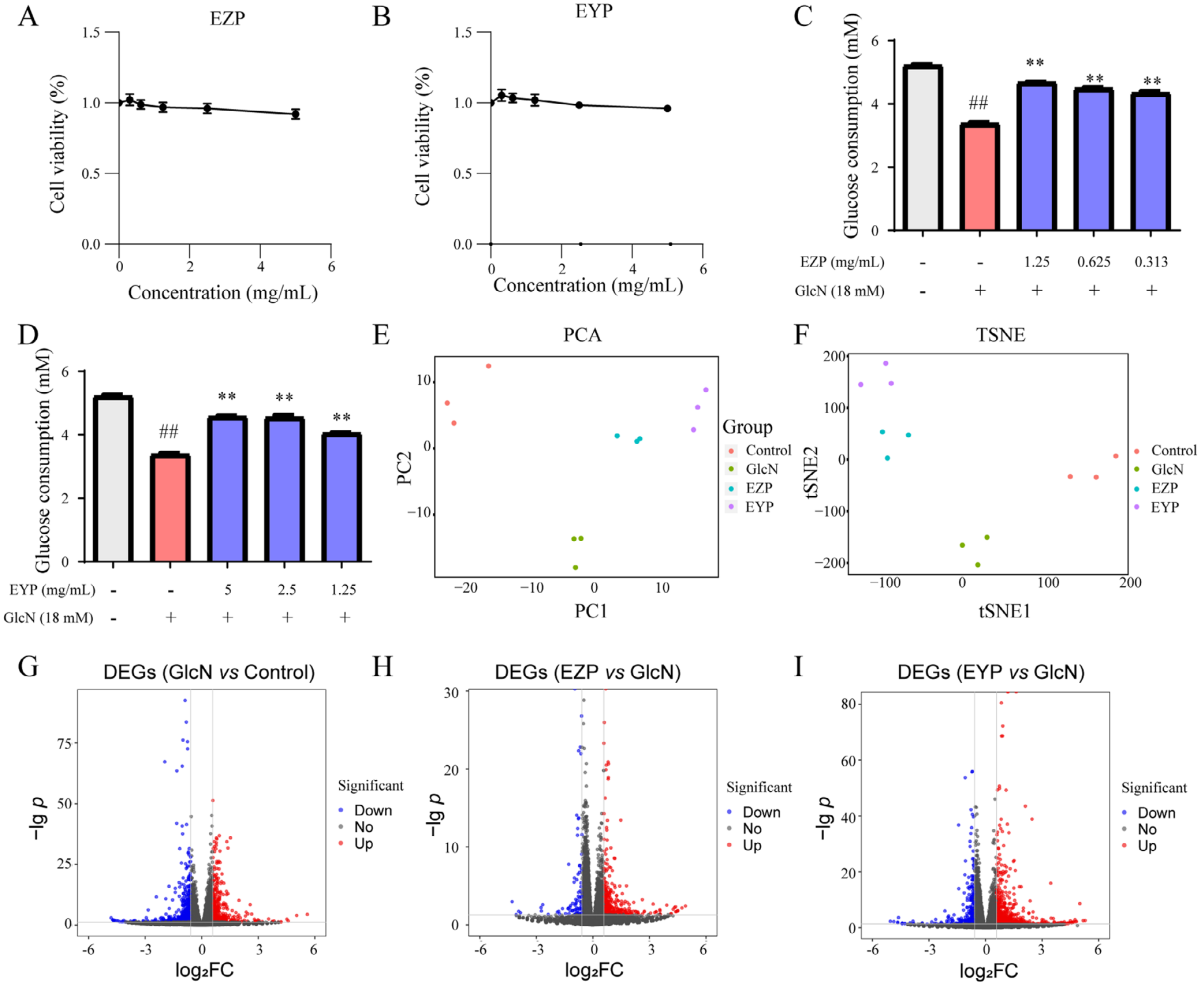
EZP and EYP reduced insulin resistance in GlcN‐induced HepG2 cells. The viability of HepG2 cells was examined following treatment with specified concentrations of EZP (A) and EYP (B) for 24 h. Glucose consumption in GlcN‐induced HepG2 was evaluated for EZP (C) and EYP (D). PCA (E) and t‐SNE (F) were conducted on the EZP and EYP groups compared to control and GlcN groups utilizing the normalized RNA‐seq read counts, respectively. Volcano plots of DEGs across various comparisons, including GlcN vs. control (G), EZP vs. GlcN (H), and EYP vs. GlcN (I) applying a cut‐off of fold change > 1.5 and *p* < 0.05 (upregulation in red and downregulation in blue).

To investigate the molecular mechanisms by which EZP and EYP alleviate diabetic nephropathy in hepatocytes, GlcN‐induced HepG2 cells were used in vitro studies. A cell viability assay assessed the cytotoxicity of EZP and EYP, which were administered at concentrations of 0.3125 to 5 mg/mL for 24 h. Results showed that EZP (Figure 3A) and EYP (Figure 3B) had minimal cytotoxic effects on HepG2 cells. The reduction in glucose consumption is a hallmark of insulin resistance. To investigate the potential roles of EZP and EYP in modulating glucose consumption, HepG2 cells were treated with GlcN in the presence of varying concentrations of EZP (0.3125 to 1.25 mg/mL) and EYP (1.25 to 5 mg/mL). Both EZP and EYP significantly enhanced the GlcN‐induced decrease in glucose consumption after 24 h (Figure 3C,D). These findings suggested that EZP and EYP effectively regulated insulin resistance.

The anti‐insulin resistance activities of EZP and EYP were further investigated through RNA‐seq analysis on HepG2 cells. The HepG2 cells (control group) were treated with extraction mimic. The GlcN‐induced HepG2 cells were treated with extraction mimic, EZP (1.25 mg/mL), or EYP (5 mg/mL) for 24 h and were named TGFβ group, EZP group, and EYP group, respectively. The GlcN group was the furthest from the control group, as evidenced by the PCA (Figure 3E) and t‐SNE (Figure 3F) analysis. Conversely, the EZP and EYP groups showed a reversal in the gene expression pattern relative to the GlcN group. Importantly, there was a close association between the clusters of the EZP or EYP group and the control group, suggesting EZP and EYP were similar to the control group in gene regulation compared with the GlcN group. These results suggested that EZP and EYP alleviated pathological changes induced by GlcN, including insulin resistance, in HepG2 cells.

### 3.4 PPARA Served as the Primary Transcriptional Factor Regulated by EZP and EYP in Insulin Resistance

The anti‐insulin resistance mechanism of EZP and EYP was analyzed using DEGs. The GlcN group had 930 DEGs compared to the control group, with 444 upregulated and 486 downregulated (Figure 3G). The EZP group had 424 DEGs compared to the GlcN group, with 340 upregulated and 84 downregulated (Figure 3H). The EYP group had 868 DEGs compared to the GlcN group, with 621 upregulated and 247 downregulated (Figure 3I).

**FIGURE 4:**
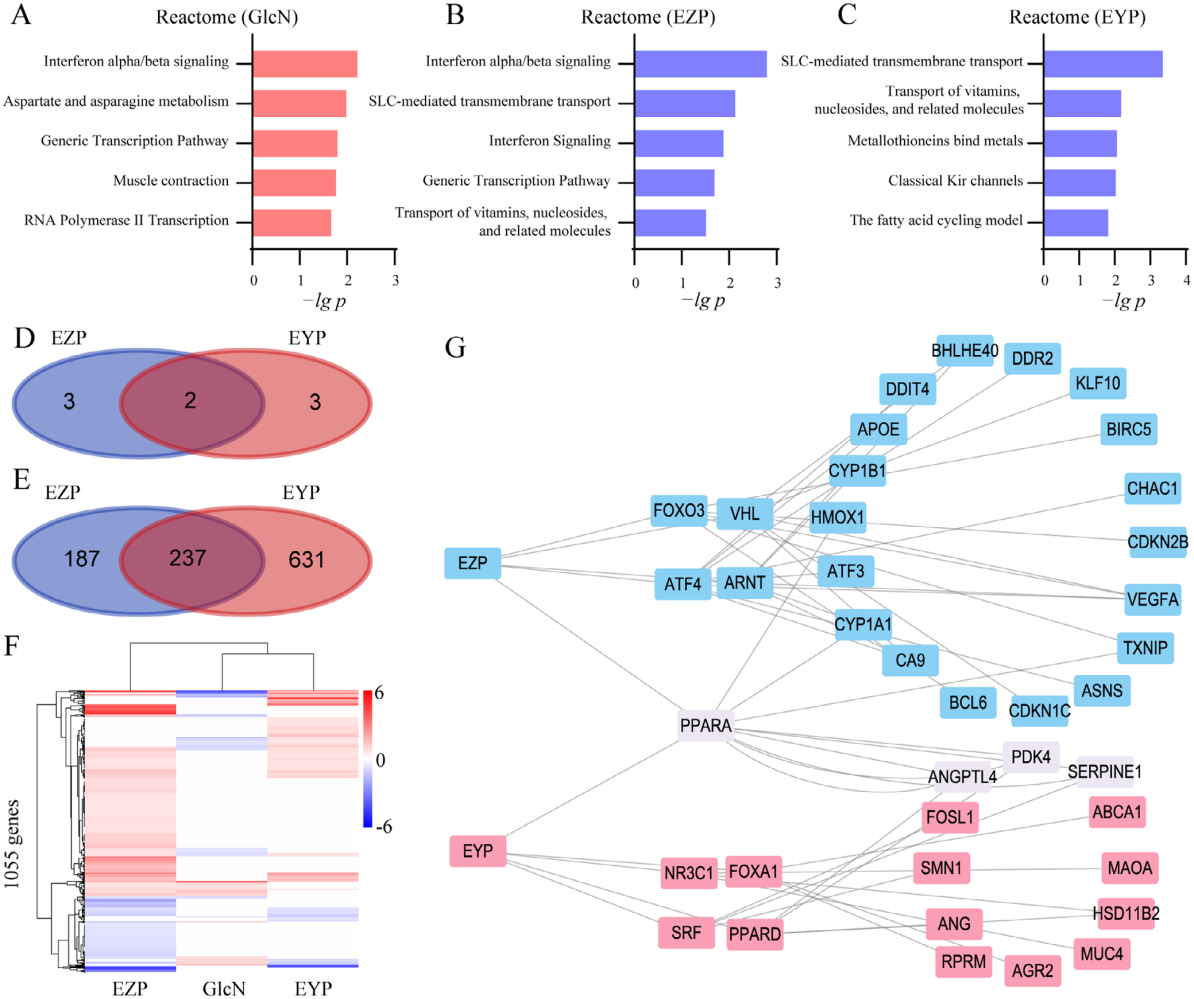
The regulation of PPARA by EZP and EYP in GlcN‐induced HepG2 cells. Pathway enrichment of GlcN (A), EZP (B), and EYP (C) group based on Reactome. Veen diagram of pathways (D) and DEGs (E) regulated by EZP and EYP. (F) Heatmap of DEGs in GlcN, EZP, and EYP. (G) transcriptional regulatory network of EZP and EYP. Transcription factors and genes regulated by EZP and EYP were highlighted in purple.

Pathway enrichment analysis revealed that GlcN significantly influenced metabolic signaling pathways, including the AGE‐RAGE signaling pathway in diabetic complications in KEGG and aspartate and asparagine metabolism in Reactome (Figure 4A and Figure S1). These findings suggested that GlcN‐induced HepG2 cells exhibited the pathological processes characteristic of metabolic abnormalities observed in diabetic nephropathy. In contrast, EZP and EYP appeared to regulate signaling pathways related to material transport, including SLC‐mediated transmembrane transport and transport of vitamins, nucleosides, and related molecules (Figure 4B,C). Only EYP showed the regulation of the fatty acid cycling model. A comparative analysis of the top five pathways and DEGs regulated by EZP and EYP revealed a relatively low proportion of overlap between the two formulae (Figure 4D,E). This finding suggested that EZP and EYP exhibited distinct regulatory mechanisms in insulin resistance cell models.

Further analysis of the DEGs pattern among GlcN, EZP, and EYP revealed a similarity despite the low intersection of DEGs between EZP and EYP (Figure 4F). To elucidate this similarity, we constructed a transcriptional regulation network for EZP and EYP. Interestingly, PPARA emerged as a transcription factor jointly regulated by EZP and EYP (Figure 4G). PPARA is a well‐established target for regulating glucose and lipid metabolism, suggesting that EZP and EYP share a common mechanism for modulating glucose and lipid metabolism in diabetic nephropathy.

### 3.5 Regulation of HIF1A Transcriptional Regulatory Network by EZP, EYP, and Active Components

**FIGURE 5:**
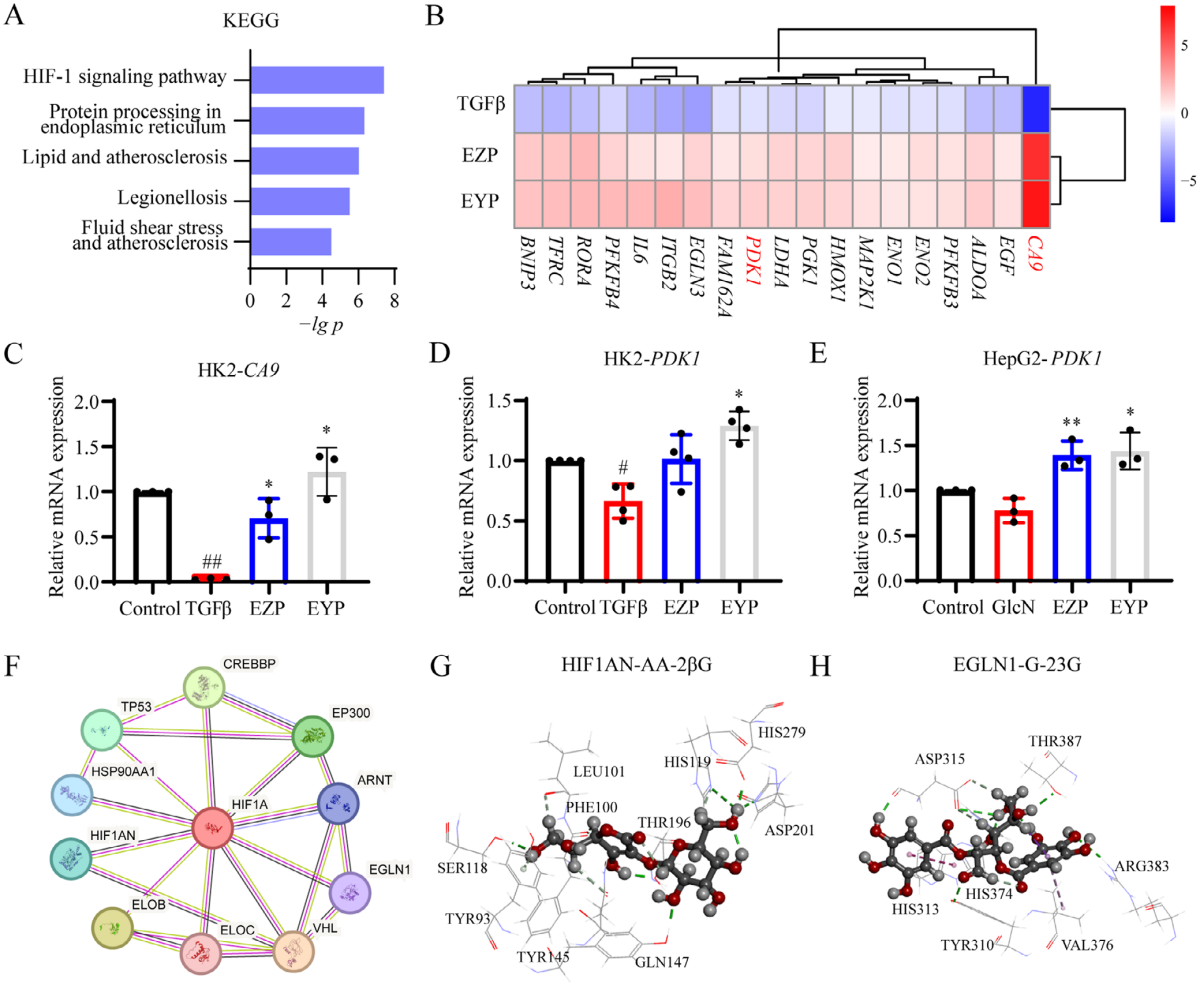
EZP, EYP, and its active components regulated transcriptional regulatory network of HIF1A. (A) Pathway enrichment of the intersection of opposite DEGs and diabetic nephropathy‐related genes. (B) Heatmap of DEGs in transcriptional regulatory network and pathways of HIF1A regulated by EZP and EYP. The qRT‐PCR of *CA9* (C) and *PDK1* (D) in TGFβ‐induced HK2 cells. (E) Gene expression of *PDK1* in GlcN‐induced HepG2 cells. (F) Protein interaction network of HIF1A. (G) Molecular docking of HIF1AN and 2‐O‐β‐d‐glucopyranosyl‐l‐ascorbic acid. (H) Molecular docking of EGLN1 and 2,3‐di‐O‐galloyl‐d‐glucose.

We investigated the material basis and mechanism of action of EZP and EYP in modulating the transcriptional regulatory network of HIF1A in HK2 and HepG2 cells. Opposite DEGs of EZP and EYP in TGFβ‐induced HK2s were first intersected with diabetic nephropathy‐related genes, and then the intersected genes were subjected to signal pathway enrichment analysis. The HIF‐1 signaling pathway emerged as the most significantly enriched signaling pathway, suggesting that it was the pivotal pathway through which EZP and EYP exerted their therapeutic effects on diabetic nephropathy (Figure 5A). The genes within the transcriptional regulatory network of HIF1A and the HIF‐1 signaling pathway were employed to analyze the regulatory mechanisms of EZP and EYP in HK2 cells. A total of 19 genes were upregulated compared to those downregulated by TGFβ in HK2 cells (Figure 5B). Among these, *CA9* exhibited the largest fold change within the transcriptional regulatory network of HIF1A, while *PDK1* was most closely associated with diabetic nephropathy.

Subsequently, qPCR was employed to validate the downstream genes of HIF1A. The expression of *CA9* was significantly downregulated by TGFβ in HK2 cells (Figure 5C) and was notably upregulated by treatment with EZP (2.5 mg/mL) and EYP (5 mg/mL). Similarly, *PDK1* expression was significantly downregulated by TGFβ and this effect was reversed by both EZP and EYP in HK2 cells (Figure 5D). Furthermore, *PDK1* was also significantly upregulated by EZP and EYP in GlcN‐induced HepG2 cells (Figure 5E).

In order to screen the active components regulating the HIF1A transcriptional regulatory network in the EZP and EYP, the PIN of HIF1A was constructed for the identification of druggable targets for the HIF1A transcriptional regulatory network (Figure 5F). HIF1AN and EGLN1 were essential druggable targets in the HIF1A transcriptional regulatory network based on PIN and literature (Ivan and Kaelin Jr. 2017). The active components were screened from EZP and EYP by molecular docking. The root mean square deviation (RMSD) of the initial ligands in the molecular docking results of HIF1AN and EGLN1 was all less than 2 Å, indicating the accuracy of the docking model (Table S2). Seven compounds were identified as the potential mediators of HIF1AN from 7 TCMs of EZP and EYP (Table S3). *Cinnamomi Cortex* and *Corni Fructus* each contained three compounds, while *Lycii Fructus* and *Eucommiae Cortex* each contained two compounds. 2‐O‐β‐d‐glucopyranosyl‐l‐ascorbic acid (AA‐2βG) with the highest score was the main active component of *Lycii Fructus* (Figure 5G). A set of 35 compounds were the potential mediators of EGLN1 (Table S4). Whereas 11 compounds were derived from *Eucommiae Cortex*, 9 compounds were derived from *Corni Fructus*. The 2,3‐di‐O‐galloyl‐d‐glucose (G‐23G) derived from *Corni Fructus* (Dong et al. 2018) achieved the highest score (Figure 5H). These results indicated that *Lycii Fructus* and *Corni Fructus*, as the common TCMs from ZGP and YGP, were essential for mediating the HIF1A transcriptional regulatory network.

### 3.6 Regulation of PPARA Transcriptional Regulatory Network by EZP, EYP, and Active Components

**FIGURE 6:**
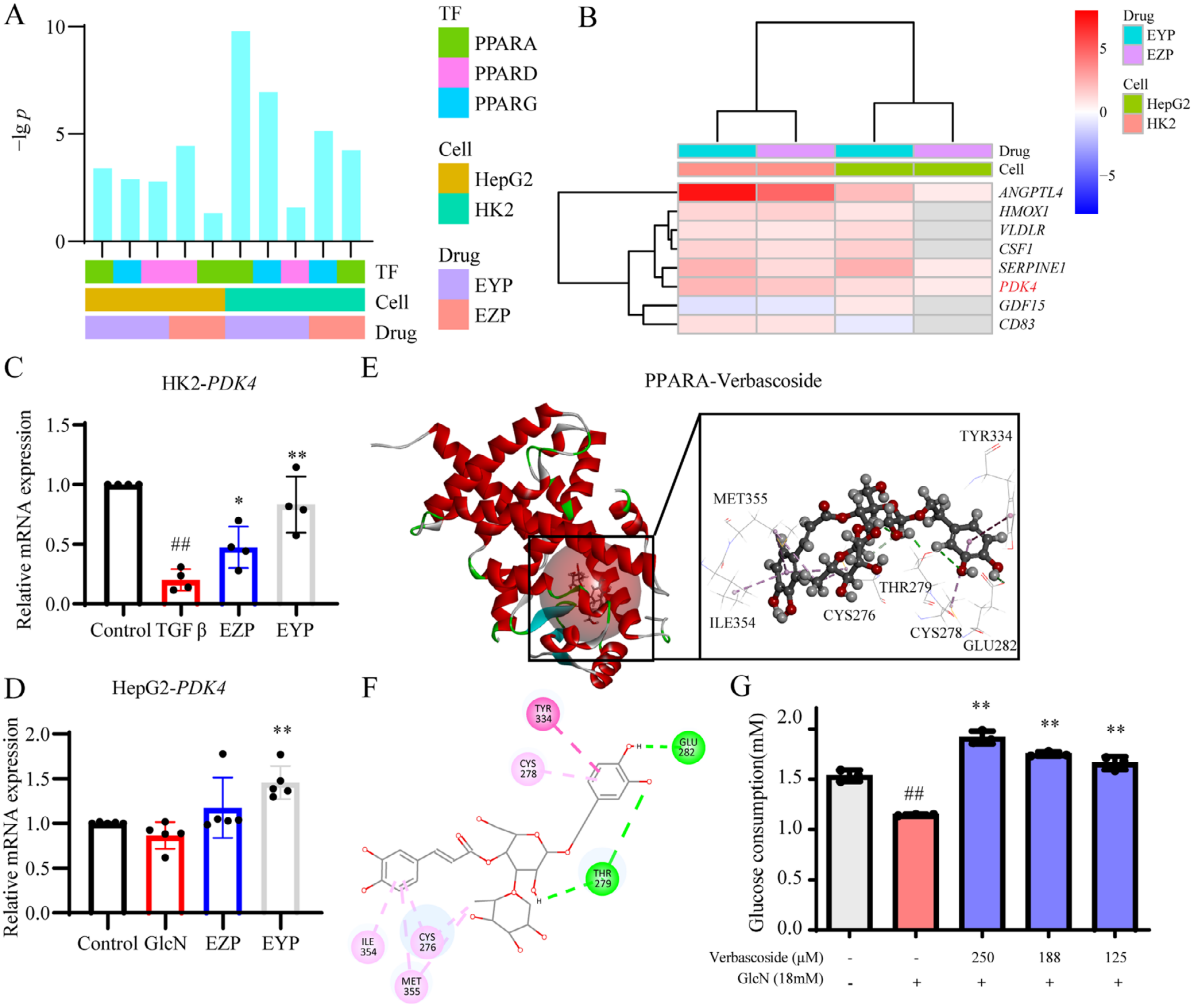
EZP, EYP, and its active components regulated transcriptional regulatory network of PPARs. (A) Enrichment of transcription factors associated with PPARs regulated by EZP and EYP in HK2 and HepG2 cells. (B) Heatmap of downstream genes in PPARA modulated by EZP and EYP in HK2 and HepG2 cells. The qRT‐PCR analysis of *PDK4* expression in TGFβ‐induced HK2 (C) and GlcN‐induced HepG2 cells (D). Three‐dimensional (E) and two‐dimensional (F) interaction graphs of verbascoside and PPARA based on molecular docking. (G) Glucose consumption assay of verbascoside in GlcN‐induced HepG2 cells.

The regulatory mechanisms of PPARs by EZP and EYP were further analyzed concerning the downstream genes and active components. The transcription factors of PPARs regulated by EZP and EYP were enriched in HK2 and HepG2 cells. Among the PPAR family members, PPARA exhibited significant enrichment in both HK2 and HepG2 cells compared to PPARG and PPARD, suggesting that PPARA is the primary transcription factor regulated by EZP and EYP (Figure 6A). Analysis of the downstream genes of PPARA revealed that eight genes showed an upregulation trend in HK2 and HepG2 cells (Figure 6B). *PDK4* was identified as a crucial gene mediating glucose metabolism and was found to be upregulated in both HK2 and HepG2 cells. The qRT‐PCR was utilized to validate the expression levels of *PDK4*. The expression of *PDK4* was significantly downregulated by TGFβ, while it was upregulated by both EZP and EYP in HK2 cells (Figure 6C). Additionally, *PDK4* expression was also upregulated in GlcN‐induced HepG2 cells (Figure 6D).

Subsequently, molecular docking was conducted to screen the active components regulating the PPARA transcriptional regulatory network from EZP and EYP. The molecular docking model for PPARA was constructed with an RMSD of the initial ligand equal to 0.5028 Å (Table S2). Eight compounds from EZP and EYP were identified as potential PPAR agonists (Table S5). Specifically, *Eucommiae Cortex* contained 4 compounds, while *Rehmanniae Radix Praeparata* and *Cuscutae Semen*, common TCMs in both EZP and EYP, contained 2 compounds each. Verbascoside, derived from *Rehmanniae Radix Praeparata*, exhibited the highest docking score among common TCMs from EZP and EYP (Figure 6E,F). The effect of verbascoside on glucose consumption was evaluated in HepG2 cells, revealing a significant increase in glucose consumption at concentrations ranging from 125 to 250 μM (Figure 6G). These results indicated that verbascoside was the main active compound in modulating the PPARA transcriptional regulatory network, thereby contributing to the therapeutic management of diabetic nephropathy.

## 4 Discussion

This study proposed a novel perspective for the treatment of diabetic nephropathy by analyzing the mechanisms of ZGP and YGP through the transcriptional regulatory networks of PPARA and HIF1A. Traditionally, PPARA has been a therapeutic target for regulating lipid metabolism and has shown promise in the treatment of diabetes and diabetic nephropathy (Hiukka et al. 2010). Additionally, PDK4, a downstream gene of PPARA, was identified as a potential key node in the gene co‐expression network of diabetic nephropathy (Han et al. 2023). However, PPARA agonists and PDK4 mediators have not been widely utilized in the clinical treatment of diabetic nephropathy. In this study, we observed that ZGP, YGP, and its components modulated PPARA and its downstream genes, PDK4, to influence renal fibrosis and glucose metabolism. These findings suggested that PPARA and PDK4 remain critical targets in the management of diabetic nephropathy.

HIF1A transcriptional regulatory networks were implicated in the pathogenesis and treatment of diabetic nephropathy. Deficiency of HIF1A has been shown to contribute to the development of diabetic nephropathy (Bohuslavova et al. 2017), whereas activation of HIF1A has been demonstrated to alleviate the condition (Tian et al. 2016). PDK1, a downstream gene of HIF1A, is a critical mediator in diabetic nephropathy, with its downregulation promoting apoptosis in renal podocytes (Wada and Makino 2016). However, there are also studies indicating that the inhibition of PDK1 significantly attenuated pulmonary fibrosis (Zhao et al. 2020). This fully reflected the double‐edged sword effect of HIF1A and its downstream pathways. CA9, another downstream gene of HIF1A, is well‐known for its association with various cancers (Giatromanolaki et al. 2020). HIF1A and CA9 were activated in TGFβ‐induced hepatoma cell line MHCC‐97H and were inhibited by sanguinarine (Su et al. 2019). Conversely, CA9 demonstrated resistance to ferroptosis under hypoxic conditions, and vanillic acid facilitated the binding of CA9 to STIM1, thereby inhibiting ferroptosis and alleviating colitis, which were consistent with the effects observed upon overexpression of CA9 (Ni et al. 2024). However, the role of CA9 in diabetic nephropathy and the regulation of renal fibrosis remains unclear. In this study, we found that ZGP, YGP, and its components activated the transcriptional regulatory networks of HIF1A, as well as the downstream genes PDK1 and CA9, which were closely associated with renal fibrosis and ECM degradation. These results indicated the activation of HIF1A‐PDK1‐CA9 was a potential novel therapeutic target for diabetic nephropathy.

Previous studies of network pharmacology have reported ZGP and YGP exerted therapeutic effects on osteoporosis by regulating the HIF‐1 pathway (Wang et al. 2024). ZGP improved ovariectomy‐induced osteoporosis by reducing PPARG levels (Li et al. 2023). In the present study, we elucidated the novel anti‐diabetic nephropathy mechanism of ZGP and YGP through PPARA and HIF1A transcriptional regulatory networks and their specific downstream genes CA9, PDK1, and PDK4. These findings offer new insights for the clinical application of these two formulae.

**FIGURE 7:**
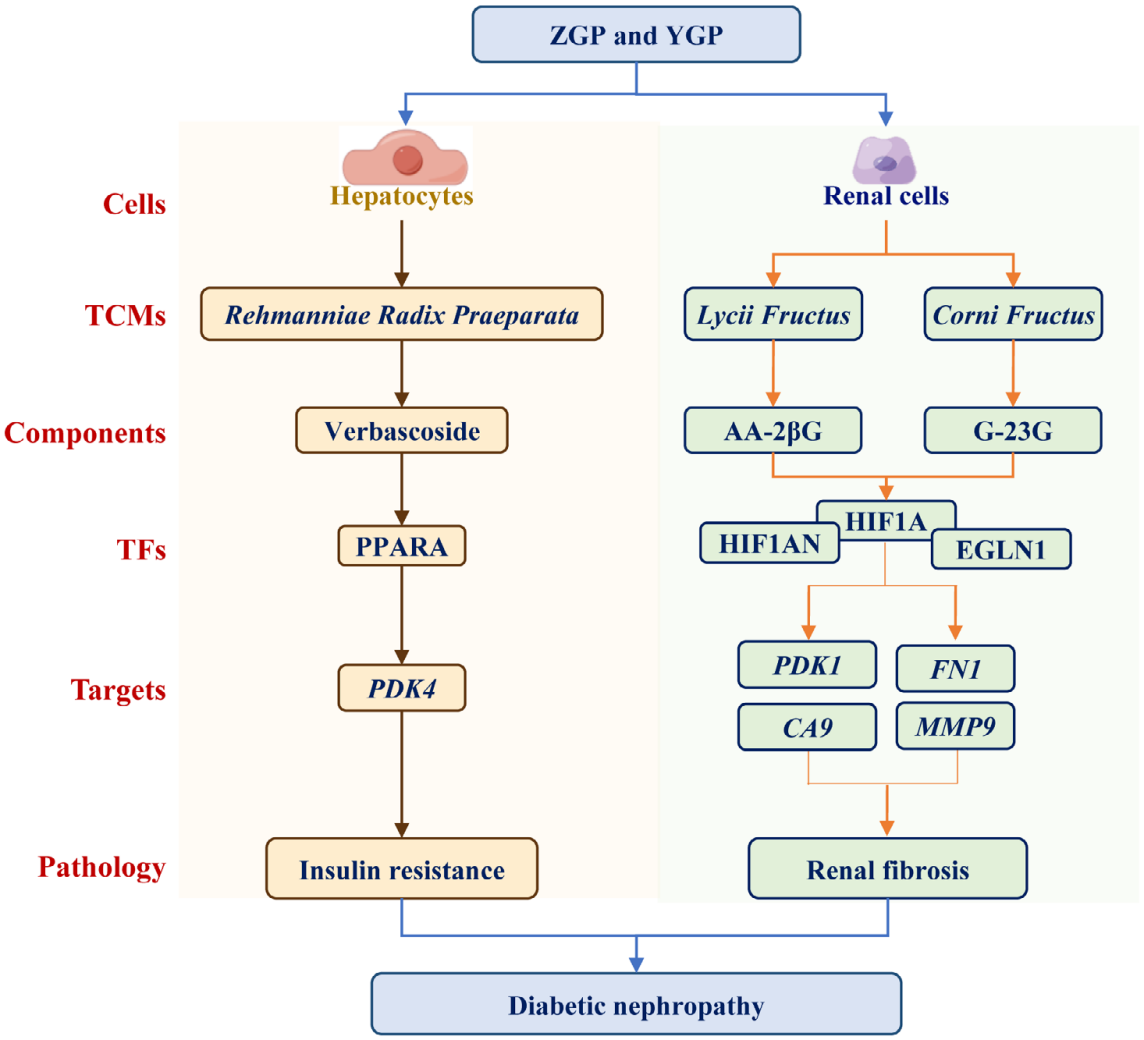
The common mechanism of ZGP and YGP for the treatment of diabetic nephropathy.

*Rehmanniae Radix Praeparata* was the root of *Rehmannia Glutinosa* and the principal TCMs in ZGP and YGP. It has demonstrated efficacy in treating diabetic nephropathy via the TGF‐β1 and Wnt/β‐catenin signaling pathways (Dai et al. 2018). Verbascoside, the main component of *Rehmanniae Radix Praeparata*, is effective in treating diabetic nephropathy through NR4A1‐LKB1‐AMPK (Chen et al. 2023) and PI3K/AKT/NF‐κB signaling pathways (Zhang, Zhang et al. 2024). Additionally, verbascoside was reported as the PPARA ligand, and its good anti‐inflammatory activity was diminished in PPARA knockout mice (Esposito et al. 2010). This study found that verbascoside from *Rehmanniae Radix Praeparata* regulated PPARA transcriptional regulatory networks and enhanced glucose consumption in HepG2 cells. These results elucidate the underlying mechanisms of verbascoside and *Rehmanniae Radix Praeparata* for treating diabetic nephropathy. The anti‐diabetic nephropathy common mechanism of ZGP and YGP is summarized in Figure 7.

Our research still had some limitations. The absorption of compounds into the blood requires further discussion for both ZGP and YGP. Future studies should also investigate other cell types associated with diabetic nephropathy, such as renal podocytes. Further analysis is needed to elucidate the mechanisms by which ZGP and YGP regulate other transcriptional regulatory networks, including SREBF1.

## 5 Conclusion

This study revealed the anti‐diabetic nephropathy mechanism of ZGP and YGP and suggested the potential targets of diabetic nephropathy through transcriptional regulatory network analysis. ZGP and YGP showed the dual roles of regulating glucose metabolism in hepatocytes and inhibiting fibrosis in renal cells. ZGP and YGP mediated HIF1A networks and downstream genes of CA9 and PDK1 in TGFβ‐induced HK2 cells for improving renal fibrosis. PPARA networks were also activated by ZGP, YGP, and their active components, including verbascoside from *Rehmanniae Radix Praeparata*, for improving fibrosis in the kidney and glucose metabolism in the liver. These results provided a new perspective for the discovery of potential targets for diabetic nephropathy and the discovery of therapeutic drugs.

## Author Contributions

**Liansheng Qiao:** methodology, writing – original draft preparation, and funding acquisition. **Xiaopeng Zhao:** validation and investigation. **Anlei Yuan:** validation. **Chaoqun Liu:** validation. **Zewen Wang:** visualization. **Xiaoqian Huo:** visualization. **Shijie Bi:** validation. **Jiaye Tian:** investigation. **Bin Yu:** investigation. **Zhaozhou Lin:** conceptualization and investigation. **Yanling Zhang:** conceptualization, writing – reviewing and editing, and funding acquisition. **Jiwang Zhang:** project administration and writing – reviewing and editing. All authors read and gave final approval to submit the manuscript.

## Conflicts of Interest

The authors declare no conflicts of interest.

## Supporting information


Appendix S1.

## Data Availability

Data will be made available on request.
