# Supplementary material for: Mechanism Analysis of Zuogui and Yougui Pills on Diabetic Nephropathy Through Transcriptional Regulatory Networks of HIF1A and PPARA
Source: Food Sci Nutr. 2025 May 23;13(6):e70317. doi: 10.1002/fsn3.70317 (PMC12102528; doi:10.1002/fsn3.70317)
Supplement: Supplementary file 1 — Appendix S1. [file FSN3-13-e70317-s001.docx]

Mechanism Analysis of Zuogui and Yougui Pill on Diabetic Nephropathy through Transcriptional Regulatory Networks of HIF1A and PPARA

Liansheng Qiao ^a*^, Xiaopeng Zhao ^a,b*^, Anlei Yuan ^a^, Chaoqun Liu ^a^, Zewen Wang ^a^, Xiaoqian Huo ^a^, Shijie Bi ^a^, Jiaye Tian ^a^, Bin Yu ^a^, Zhaozhou Lin ^a,b^, Yanling Zhang ^a, †^, Jiwang Zhang ^a, †^

a Key Laboratory of TCM-information Engineer of State Administration of TCM, School of Chinese Materia Medica, Beijing University of Chinese Medicine, Beijing 102488, China

b Beijing Tong Ren Tang Technology Development Co., Ltd., Beijing 100079, China

^*^ These authors contributed equally to this work.

^†^ Correspondence: zhangyanling@bucm.edu.cn (Y. Zhang), zhangjiwangbzy@163.com (J. Zhang)

## Supplementary data


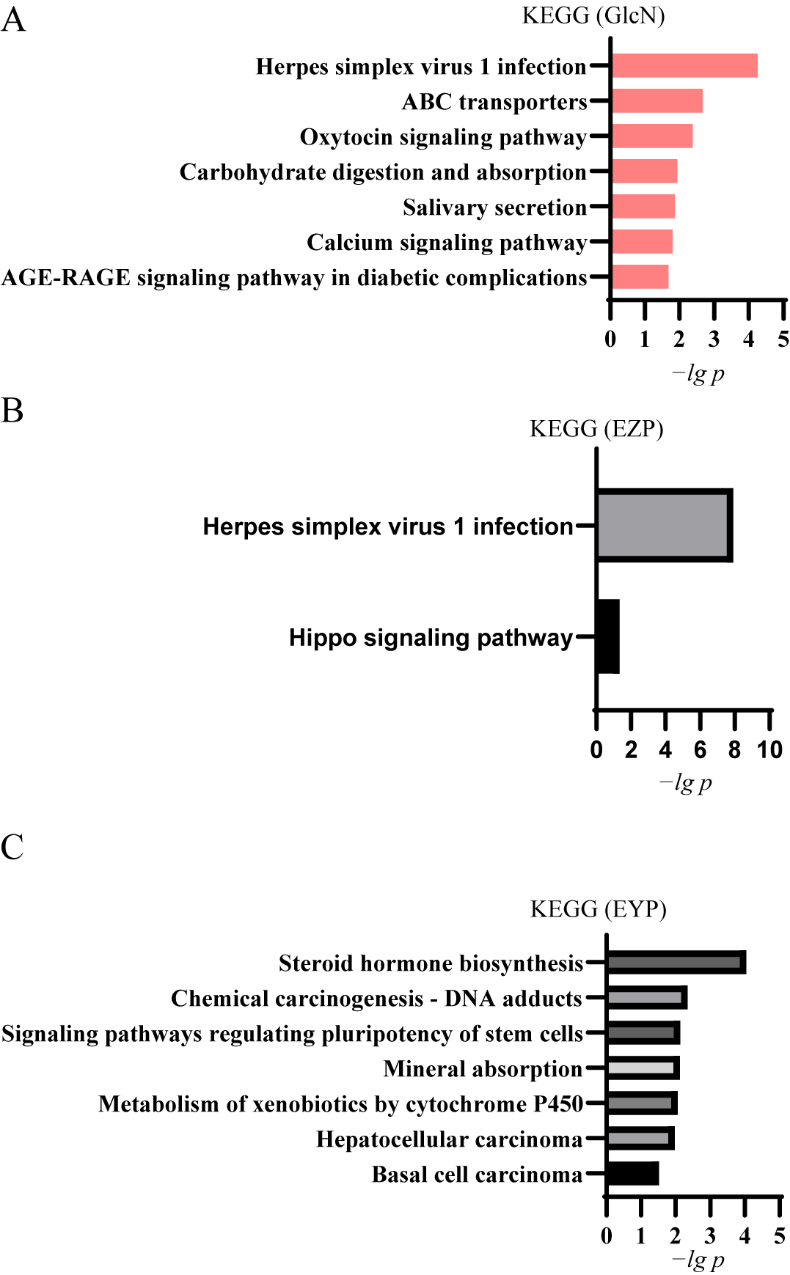


Figure S1 Pathway enrichment of EZP and EYP in GlcN-induced HepG2 cells based on KEGG. (A) KEGG pathway enrichment of GlcN. (B) KEGG pathway enrichment of EZP. (C) KEGG pathway enrichment of EYP group.

Table S1 List of primer sequences of human origin used in this study

| Primer names | F sequences (5’ – 3’) | R sequences (5’ – 3’) |
| --- | --- | --- |
| *FN1* | GGCTTGAACCAACCTACGGATGAC | TCCTTCTGCCACTGTTCTCCTACG |
| *CA9* | CTGTCTCGCTTGGAAGAAATCG | GCAGGAGTGCAGATATGTCCAG |
| *MMP9* | AGTCCACCCTTGTGCTCTTCCC | TCTCTGCCACCCGAGTGTAACC |
| *PDK1* | CTGTGATACGGATCAGAAACCG | TCCACCAAACAATAAAGAGTGCT |
| *PDK4* | GGAGCATTTCTCGCGCTACA | ACAGGCAATTCTTGTCGCAAA |
| *GAPDH* | GGAGCGAGATCCCTCCAAAAT | GGCTGTTGTCATACTTCTCATGG |

Table S2 Molecular docking models of HIF1AN (1H2M), EGLN1 (4BQW), and PPARA (3KDU)

| PDB ID | RMSD (Å) | -CDOCKER interaction energy of initial ligands (kcal/mol) |
| --- | --- | --- |
| 1H2M | 0.4512 | 46.8321 |
| 4BQW | 0.7509 | 53.0739 |
| 3KDU | 0.5028 | 73.4724 |

Table S3 The potential active compounds acting on HIF1AN from Zuogui Pill and Yougui Pill based on molecular docking

| Name | Pubchem ID | -CDOCKER interaction energy of initial ligands (kcal/mol) | TCMs |
| --- | --- | --- | --- |
| 2-o-beta-d-glucopyranosyl-l-ascorbic acid | 54706833 | 48.8648 | Gouqizi |
| nonanedioate | 3801344 | 48.5399 | Shanzhuyu, Danggui |
| (2S)-2-azaniumyl-6-(carbamoylamino)hexanoate | 6991977 | 48.2476 | Duzhong |
| n-hexanol | 57349101 | 45.9428 | Rougui |
| magnesium | 18705522 | 44.5499 | Shanzhuyu |
| Heptanoate | 93052 | 42.5333 | Rougui |
| 3,4-dihydroxybenzoate | 54675866 | 42.3537 | Gouqizi, Shanzhuyu, Rougui, Duzhong |

Table S4 The potential active compounds acting on EGLN1 from Zuogui Pill and Yougui Pill based on molecular docking

| Pubchem ID | Name | -CDOCKER interaction energy of initial ligands (kcal/mol) | TCMs |
| --- | --- | --- | --- |
| 2,3-di-O-galloyl--d-glucose | 21146989 | 57.3776 | Shanzhuyu |
| Procyanidin B5 | 124017 | 57.0182 | Rougui |
| SILYMARIN | 5213 | 56.8467 | Gouqizi |
| Isocarlinoside | 73802481 | 56.4231 | Tusizi |
| Isomangiferin | 5318597 | 56.4174 | Duzhong |
| L-Octanoylcarnitine | 11953814 | 56.1989 | Rougui |
| Eucommin A | 442836 | 56.0231 | Duzhong |
| Genipin 1-gentiobioside | 3082301 | 54.2323 | Duzhong |
| (E)-Ferulate | 54691413 | 53.8196 | Gouqizi |
| Fuzinoside | NA | 53.72 | Fuzi |
| Olivil 4'-O-glucoside | 14033815 | 52.6516 | Duzhong |
| RAFFINOSE | 439242 | 52.515 | Shudihuang |
| Cistanoside F | 101688189 | 51.4774 | Shudihuang |
| (2S)-2-[[4-[(2-amino-4-oxo-1H-pteridin-6-yl) methylamino]benzoyl]amino]pentanedioic acid | 6037 | 50.7715 | Danggui |
| 4-(phosphooxymethyl)-2-furancarboxaldehyde(2-) | 25201088 | 50.5636 | Shanzhuyu |
| Puerarin | 5281807 | 50.4418 | Chuanniuxi |
| ALBASPIDIN | 42738 | 50.4221 | Duzhong |
| Ipolamiide | 442425 | 50.4052 | Gouqizi |
| Isoquercetin | 5280804 | 49.9975 | Shanzhuyu, Duzhong |
| methyl (2S,3R,4R)-3-ethenyl-4-[2-(3,4,5-trihydroxybenzoyl)oxyethyl]-2-[(2S,3R,4S,5S,6R)-3,4,5-trihydroxy-6-(hydroxymethyl)oxan-2-yl]oxy-3,4-dihydro-2H-pyran-5-carboxylate | 122173200 | 49.9577 | Shanzhuyu |
| 8-O-Acetylharpagide | 9978650 | 49.8764 | Duzhong |
| Rehmannioside A | 101787412 | 49.8076 | Shudihuang |
| Verbenalin | 73467 | 49.7948 | Shanzhuyu |
| methyl (4aR,7aS)-7-(hydroxymethyl)-1-[3,4,5-trihydroxy-6-(hydroxymethyl)oxan-2-yl]oxy-1,4a,5,7a-tetrahydrocyclopenta[c]pyran-4-carboxylate | 137706142 | 49.5008 | Shudihuang, Duzhong |
| Cacticin | 5318644 | 48.8829 | Shanzhuyu |
| Puerarin | 53384442 | 48.804 | Chuanniuxi |
| Morroniside | 138107798 | 48.7672 | Shanzhuyu |
| methyl 7-methyl-5-oxo-1-[3,4,5-trihydroxy-6-(hydroxymethyl)oxan-2-yl]oxy-4a,6,7,7a-tetrahydro-1H-cyclopenta[c]pyran-4-carboxylate | 273110 | 48.6942 | Shanzhuyu |
| linoleic acid | 5867807 | 48.55 | Rougui |
| 7-(8-formyl-1,7-dihydroxy-6-methoxy-3-methyl-5-propan-2-ylnaphthalen-2-yl)-2,8-dihydroxy-3-methoxy-6-methyl-4-propan-2-ylnaphthalene-1-carbaldehyde | 375713 | 48.5066 | Duzhong |
| ISOMALTOSE | 133612057 | 48.3409 | Duzhong |
| n-hexanol | 57349101 | 48.258 | Rougui |
| (-)-Epicatechin gallate | 107905 | 48.1675 | Shanzhuyu, Rougui |
| GARCINONE D | 5495926 | 47.8794 | Shanyao |
| Acanthoside B | 443024 | 47.7793 | Duzhong |

Table S5 The potential active compounds acting on PPARA from Zuogui Pill and Yougui Pill based on molecular docking

| Name | Pubchem ID | -CDOCKER interaction energy of initial ligands (kcal/mol) | TCMs |
| --- | --- | --- | --- |
| OCTACOSANOIC ACID | 10470 | 70.5479 | Duzhong |
| Verbascoside | 5281800 | 70.3186 | Shudihuang |
| cerotate | 5461023 | 69.1566 | Tusizi |
| RUTIN | 5280805 | 68.111 | Gouqizi, Shanzhuyu, Tusizi, Duzhong |
| 1,3,6-tris-o-(3,4,5-trihydroxybenzoyl)hexopyranose | 250395 | 67.7817 | Shudihuang |
| Acanthoside B | 443024 | 66.4825 | Duzhong |
| Metarene | 15561185 | 66.3978 | Shanyao |
| Lignoceric acid | 11197 | 66.2769 | Danggui, Duzhong |
